# Kinin and Purine Signaling Contributes to Neuroblastoma Metastasis

**DOI:** 10.3389/fphar.2018.00500

**Published:** 2018-05-18

**Authors:** Henning Ulrich, Mariusz Z. Ratajczak, Gabriela Schneider, Elena Adinolfi, Elisa Orioli, Enéas G. Ferrazoli, Talita Glaser, Juliana Corrêa-Velloso, Poliana C. M. Martins, Fernanda Coutinho, Ana P. J. Santos, Micheli M. Pillat, Ulrich Sack, Claudiana Lameu

**Affiliations:** ^1^Departamento de Bioquímica, Instituto de Química, Universidade de São Paulo, São Paulo, Brazil; ^2^James Graham Brown Cancer Center, Stem Cell Institute, University of Louisville, Louisville, KY, United States; ^3^Department of Regenerative Medicine, Center for Preclinical Research and Technology, Warsaw Medical University, Warsaw, Poland; ^4^Section of Pathology, Oncology and Experimental Biology, Department of Morphology, Surgery and Experimental Medicine, University of Ferrara, Ferrara, Italy; ^5^Institute of Clinical Immunology, Universitätsklinikum Leipzig, Leipzig, Germany

**Keywords:** metastasis, bradykinin, neuroblastoma, purinergic system, bone marrow

## Abstract

Bone marrow metastasis occurs in approximately 350,000 patients that annually die in the U.S. alone. In view of the importance of tumor cell migration into the bone marrow, we have here investigated effects of various concentrations of stromal cell-derived factor-1 (SDF-1), bradykinin- and ATP on bone marrow metastasis. We show for first time that bradykinin augmented chemotactic responsiveness of neuroblastoma cells to SDF-1 and ATP concentrations, encountered under physiological conditions. Bradykinin upregulated VEGF expression, increased metalloproteinase activity and induced adhesion of neuroblastoma cells. Bradykinin augmented SDF-1-induced intracellular Ca^2+^ mobilization as well as resensitization and expression of ATP-sensing P2X7 receptors. Bradykinin treatment resulted in higher gene expression levels of the truncated P2X7B receptor compared to those of the P2X7A full-length isoform. Bradykinin as pro-metastatic factor induced tumor proliferation that was significantly decreased by P2X7 receptor antagonists; however, the peptide did not enhance cell death nor P2X7A receptor-related pore activity, promoting neuroblastoma growth. Furthermore, immunodeficient nude/nude mice transplanted with bradykinin-pretreated neuroblastoma cells revealed significantly higher metastasis rates compared to animals injected with untreated cells. In contrast, animals receiving Brilliant Blue G, a P2X7 receptor antagonist, did not show any specific dissemination of neuroblastoma cells to the bone marrow and liver, and metastasis rates were drastically reduced. Our data suggests correlated actions of kinins and purines in neuroblastoma dissemination, providing novel avenues for clinic research in preventing metastasis.

## Introduction

Neuroblastoma is the most common pediatric extracranial solid tumor and present as metastatic disease in about 50% of patients (Kucia et al., 2005a). The bone marrow (BM) is the main site of metastasis in advanced neuroblastoma, and detection of minimal residual neuroblastoma cells at this site correlates with poor prognosis (Seeger et al., 2000). The identification of new therapeutic targets causing neuroblastoma metastatic dissemination is of particular interest for advanced stage neuroblastoma patients, which despite aggressive treatment still show overall survival rate less than 40%. Although chemo- and radiotherapy are the most important and effective therapeutic modalities used in cancer treatment, these are associated with undesired expression and release of several pro-metastatic factors, such as molecules related to inflammatory processes creating a favorable microenvironment for seeding of tumor cells that have survived treatment (Ratajczak et al., 2013).

The C-X-C chemokine receptor type 4 (CXCR4) and its ligand, α-chemokine stromal-derived factor-1 (SDF-1), secreted by BM stroma supposedly participate in establishment of BM metastasis by neuroblastoma cells (Geminder et al., 2001). However, SDF-1 dose-dependent migration of neuroblastoma was observed only when CXCR4 expression and activity had been upregulated. Thus, we here propose the existence of other mechanisms regulating neuroblastoma trafficking to the BM and/or increasing responsiveness of neuroblastoma to SDF-1 by sensitization or overexpression of the CXCR4.

For this, we have studied interactions of factors originated by inflammatory processes and/or leakage of damaged cells, such as adenosine triphosphate (ATP) and bradykinin (BK) (Pinheiro et al., 2013), regarding their priming effects on responsiveness to low concentration of SDF-1 and motility and invasiveness properties of neuroblastoma cells for metastasis formation.

BK, produced by the kallikrein–kinin system, is implicated in many pathophysiological processes including tumor proliferation, migration, angiogenesis, and increased vascular permeability (da Costa et al., 2014), and, therefore, probably contributing to the metastatic behavior of neuroblastoma tumors. Extracellular ATP participates in important steps of metastasis, including rearrangement of cytoskeleton, invasion, migration and adhesion (Schneider et al., 2015). Extracellular ATP activates plasma membrane purinergic P2 receptors, subdivided into P2X ligand-gated ion channels, formed as homo- or hetero-trimeric receptors by assembly of P2X1-P2X7 subunits, and P2Y G protein-coupled receptors (P2Y1, P2Y2, P2Y4, P2Y6, P2Y11, P2Y12, and P2Y14) (Burnstock, 2007).

Among other P2 receptors, the P2X7 subtype recently attracted increasing attention regarding its role in carcinogenesis (Adinolfi et al., 2015; Di Virgilio et al., 2016). This receptor covers a dual role in tumor cells exerting a tonic proliferative activity at low concentrations of agonist and behaving as pro-apoptotic receptor at milimolar concentrations of ATP (Adinolfi et al., 2005). Of interest, the known functional splice variants of the P2X7 receptor, i.e., P2X7A and B isoforms, have been associated with proliferation and cancer progression or cytotoxicity (Adinolfi et al., 2010; Giuliani et al., 2014).

We have focused on biological responses to stimulation of exogenously administered BK on neuroblastoma cell lines, such as chemotaxis in response to SDF-1 and ATP, expression and sensitization of CXCR4 and purinergic receptors, ATP-induced pore formation, cell proliferation, adhesion, MMPs activities, VEGF expression and cytoskeleton rearrangement *in vitro*, as well as induction of neuroblastoma cell dissemination *in vivo*. Altogether, our findings provide proof for interactions of BK with the purinergic signaling system and also with the CXCR4-SDF-1 axis involved in metastasis, making them attractive targets for development of anti-cancer drugs.

## Materials and methods

### Radiation of mice and isolation of bone marrow cells

Animal experimentation and sacrifice followed a protocol approved by the Ethics' committee of the University of Louisville and regulations established by the NIH. Expression patterns of kininogens 1 and 2 and BK levels in the BM were obtained after 24 h of exposing mice C57BL/6 to a lethal dose of γ-irradiation (1,000 cGy) by real-time PCR using the primer sequences listed in Table 1 and an ELISA kit, respectively. BM cell lysates were obtained by flushing the BM of tibia and femur cavities, and resuspending the cells of each BM in 3 ml of RPMI medium, then incubated for 1h at 37°C and centrifuged (680 × g, 10 min, 4°C). Red blood cells from the BM were then lysed using “Lysis Buffer” (Becton & Dickinson, San Jose, CA) and the white blood cells pellet were stored at −80°C for later use in the quantification protocol.

**Table 1:** Primer sequences.

**Gene**	**Forward primer (5′-3′)**	**Reverse primer (5′-3′)**	**Species**
P2X1	TTGTGGAGAACGGGACCAA	GTCAAAGCGAATCCCAAACAC	*Homo sapiens*
P2X2	GATCCGCATTGACGTCATTG	TGGTGGGAATCAGGCTGAAC	*Homo sapiens*
P2X3	GCATCCCCAAATACTCCTTCAC	GGACACGCTGCTTTTCTCAGA	*Homo sapiens*
P2X4	GCCGCCTCGATACACGGGAC	TGCTCGTTGCCAGCCAGGTC	*Homo sapiens*
P2X5	CCTTCCTGCCAGCTGTTTG	TGCCATCTCCCCCACTTTAA	*Homo sapiens*
P2X6	CCAAACAACACCACCGAGATC	TGGGACCAAGAGGAGAATTCC	*Homo sapiens*
P2X7	ACTGCAACCTAGACCGTTGGTT	TCAAGGCGACGGAAACTGTAT	*Homo sapiens*
P2X7A	CGGCTCAACCCTCTCCTACT	GGAGTAAGTGTCGATGAGGAAGTC	*Homo sapiens*
P2X7B	GGAAAATGGTTTGGAGAAGGAAGTG	CGATGAGGAAGTCGATGAACACA	*Homo sapiens*
P2Y1	GGATGCCATGTGTAAACTGC	GTACACCACACCGCTGTACC	*Homo sapiens*
P2Y2	CACCCGCACCCTCTACTACT	CCTTGTAGGCCATGTTGATG	*Homo sapiens*
P2Y4	CCGTCCTGTGCCATGACA	GCTGAAGTGCACATAGTGGTCAA	*Homo sapiens*
P2Y6	GCCGGCGACCACATGA	CCTGCCTCTGCCATTTGG	*Homo sapiens*
P2Y11	CATGGCAGCCAACGTCTCGG	GGGCCACAGGAAGTCCCCCT	*Homo sapiens*
P2Y12	AATGCAAGCCGTCGACAAC	CTCTGGTGCACAGACTGGTGTT	*Homo sapiens*
P2Y13	GTGCCACGAGCTCCAACAC	TGAGGCCATGGAAGAAAACG	*Homo sapiens*
P2Y14	TCTTCATTGCAGGAATCCTACTCA	AGAGCTGGGCACGTAAAAGAAT	*Homo sapiens*
Kininogen 1	AGGGCAACTGCTCTGCTCAG	TGAAGTCACAGTCCTGCCATG	*Mus musculus*
Kininogen 2	AGGCTGTGGATGCCTCTCTG	TGCCGCTTTGTAACCTAGCA	*Mus musculus*
β2-microglobulin	AATGCGGCATCTTCAAACCT	TGACTTTGTCACAGCCCAAGATA	*Homo sapiens*
GAPDH	CCTCCCGCTTCGCTCTCT	GCTGGCGACGCAAAAGA	*Homo sapiens*
GAPDH	TGCACCACCAACTGCTTAG	GGATGCAGGGATGATGTTC	*Mus musculus*
VEGF	CACCCATGGCAGAAGGAGGA	GGTCTCGATTGGATGGCAGTAG	*Homo sapiens*
MMP2	TCAAGGGCATTCAGGAGCTC	TGCCAAGGTCAATGTCAGGAG	*Homo sapiens*
MMP9	TGGTTACACTCGGGTGGCA	GGCCCCAGAGATTTCGACTC	*Homo sapiens*
α-satellite	ACCACTCTGTGTCCTTCGTTCG	ACTGCGCTCTCAAAAGGAGTGT	*Homo sapiens*
β-actin	TTCAATTCCAACACTGTCCTGTCT	CTGTGGAGTGACTAAATGGAAACC	*Mus musculus*

### Quantification of BK by ELISA

BK levels of BM cell lysates of six irradiated C57/BL6 mice and six non-irradiated mice (control) were measured using the Bradykinin (Human, Rat, Mouse) ELISA Kit (Phoenix Pharmaceuticals, Inc., Burlingame, CA), according to the manufacturer's instructions. Briefly, 50 μL of standard solutions or test samples were added to immunoplate multiwells, then 25 μL of each primary antisera and biotinylated peptide solution were added, and the plates were incubated for 2 h at room temperature with mild agitation. The plates were washed 5 times, and 100 μL of diluted streptavidin-conjugated horse radish peroxidase solution was added to each well. After 60 min incubation at room temperature, immunoplates were washed 5 times, and 100 μL of 3, 3′, 5, 5′-tetramethyl benzidine.diHCl (TMB) solution was added to each well. After further 20 min incubation at room temperature, the reaction was stopped with 100 μL of 2N HCl. The absorbance was measured at 450 nm using the FlexStation III microplate reader (Molecular Devices, Sunnyvale, CA). TMB solution and 2N HCl (1:1) were used as a blank control.

### Cell lines

Cells were cultured in appropriate media at 37°C in 5% CO_2_ atmosphere. The medium was changed every 2 days. CHP-100 and CHP 134 human neuroblastoma cell lines were maintained in RPMI-1640, while IMR-32 and SH-SY5Y cell lines were kept in Minimum Essential Medium and Dulbecco's Modified Eagle's Medium, respectively, all of them containing 10% FBS, 100U/mL penicillin and 10 μg/mL streptomycin. Both, CHP-100 (ECACC; 06122001) and SH-5YSY (CRL-2266) lines, are neuroblastoma cells isolated from metastatic site: bone marrow. The CHP-134 cell line (ECACC; 06122002) was obtained from the primary neuroblastoma tumor mass of the adrenal gland, and IMR-32 cells (CCL-127) were derived from metastatic sites in the abdominal mass.

### Adhesion assay

Neuroblastoma cells were maintained in a quiescent state for 24 h in medium supplemented with 0.2% BSA in the absence or presence of BK (10, 30, or 1,000 nM). Cells (5 × 10^4^/well) were directly added to wells pretreated with 10 μg/mL fibronectin at 4°C overnight and blocked with 0.5% BSA for 2 h before the experiment. After 8 min incubation at 37°C, 96-well plates were vigorously washed three-times to remove nonadherent cells. Adherent cells were counted using an inverted microscope.

### Immunofluorescence assay

Immunocytochemistry assays were performed according to Lameu et al. (2012). Briefly, the CHP-100 cells were fixed with 4% para-formaldehyde (PFA), permeabilized and blocked during 1 h with 3% BSA and 0.25% (v/v) Triton X-100 in PBS, followed by an overnight incubation with the anti-Vascular Endothelial Growth Factor primary antibody (V6627, Anti-VEGF, Sigma-Aldrich) at final concentration of 10 μg/mL. After that, cells were washed with PBS, and secondary anti-Goat Cy3 (C2821, Sigma) at 1:100 dilution were added and incubated for 3h. Sequentially, washing with PBS 1x and DAPI (CALBIOChem; 1 μg/mL) was used as a nuclear stain. Slides were mounted and analyzed under a fluorescence microscope (Axiovert 200, Zeiss, Jena, Germany) and analyzed with Image J (Wayne Rasband, NIH, Bethesda, MD).

### Gelatin zymography

To evaluate MMP-2 and MMP-9 activities, CHP-100 cells were incubated for 24h in serum-free medium in the absence or presence of 10 nM BK, and zymography was carried out as described previously (Jankowski et al., 2003) using 200 μg total protein of cell lysate obtained with EDTA-free RIPA. Quantitative assessment of active form MMP-2 band intensity was done by densitometry using program Image J software (Wayne Rasband, NIH, Bethesda, MD).

### Analysis of rearrangements of actin cytoskeleton

To visualize the actin cytoskeleton, CHP-100 cells were cultured in RPMI 1640 with 10% FBS on slides with chambers until appropriate attachment. Then, the medium was changed and the cells were maintained in serum-free media in the absence or presence 10 nM BK for 24 h. Subsequently, the cells were fixed for 30 min in 4% paraformaldehyde/PBS and then stained for 1 h with Alexa Fluor 488 Phalloidin (Cell Signaling, Danvers, MA) at a concentration of 330 nM. Images were obtained with a fluorescence microscope (Axiovert 200, Zeiss, Jena, Germany) and analyzed with the StrataQuest software (TissueGnostics GmbH, Vienna, Austria). Briefly, the StrataQuest software is part of the TissueFAXS™ Cytometry platform—a means of image cytometry of tissue sections, tissue- and cell-cultures with a workflow and data display similar to flow cytometry. The software provides cellular data in dot-plots to show multiple measurement parameters of single cells, multicellular structures and/or morphological features.

### Chemotaxis assay

Polycarbonate membranes with 8 μm pores were treated with 50 μl of 0.5% gelatin. Cells were trypsinized, washed and re-suspended in medium without serum containing 0.2% BSA and seeded into the upper chamber of a transwell insert (Costar Transwell, Costar Corning, Corning NY) at a density of 3 × 10^4^ at 120 μl. The incubation period of drugs for primed cells was 3 h for ATP and 24 h for BK. The bottom part was filled with the potential chemotactic agents or 0.2% BSA (control). After 24 h, the inserts were removed from the transwell. In case of the involvement of gene expression, a period of 24 h should be enough for observing effects on chemotaxis. The cells remaining in the upper chamber were removed with a cotton swab, while those that transmigrated were stained with HEMA 3 (Fisher Scientific) and counted using an inverted microscope.

### Flow cytometry analysis

The expression of CXCR4 by neuroblastoma cell lines was detected with an Allophycocyanin (APC)-anti-CXCR4 monoclonal antibody (Becton & Dickinson), clone no. 12.G5. Briefly, the cells were stained in phosphate-buffered saline (PBS; Ca- and Mg-free) supplemented with 2% bovine calf serum (BCS; Hyclone, Logan, UT). After the final wash, cells were resuspended in PBS and analyzed by flow cytometry using the LSRII (Becton Dickinson, San Jose, CA).

### Real time polymerase chain reaction (qPCR)

Gene expression levels were determined by real-time PCR, as previously described (Lameu et al., 2012). From the total RNA extracted of neuroblastoma cells, cDNA strands were synthetized using the cDNA Cycle Kit (Thermofisher), following manufacturer's recommendations. Real time PCR was performed in 25 μl of buffer reaction containing 1 μl of cDNA, SYBR Green Master Mix (Thermofisher), and 5 pmol of each sequence-specific primer (Table 1). Thermal cycling carried out with the ABI Step One Plus instrument (Thermofisher) consisted of a pre-incubation step for 2 min at 50°C, then denaturation for 10 min at 95°C followed by 40 cycles for denaturation for 15 s at 95°C, and annealing/extension for 1 min at 60°C. Gene expression levels of GAPDH (glyceraldehyde-3-phosphate dehydrogenase) were used for normalization of gene expression. Relative gene expression of P2X7A and P2X7B receptor isoforms were determined with the TaqMan assay as previously described (Adinolfi et al., 2010). Gene expression levels were normalized by using β2-microglobulin as endogenous control (for primer sequences see Table 1). The results were analyzed for relative quantitation among groups using the comparative 2^−ΔΔCT^ method (Livak and Schmittgen, 2001).

### Calcium imaging in single cells

Forty-eight hours before calcium measurements, 2.5 × 10^5^ cells were seeded and allowed to attach on p35-mm culture dishes (Nalge Nunc International, Rochester, NY). Cells were loaded for 30 min at 37°C with 5 μM Fluo-3AM (Sigma Chemical, St. Louis, MO) in the presence of 0.5% Me_2_SO and 0.1% of the nonionic surfactant pluronic acid F-127, followed by three washes with DMEM containing 10% FBS. Fluo-3 fluorescence was excited with a xenon lamp at 488 nm, and the emitted light was detected using a bandpass filter at 515–530 nm. Measurements of transient changes in free intracellular Ca^2+^ concentration [Ca^2+^]_*i*_ in neuroblastoma cells were evaluated by calcium imaging with an inverted Microscope (ECLIPSE-TiS, Nikon, Melville, NY), equipped with a 14 bit high-resolution CCD camera (Cool-SNAP HQ2, Photometrics, Tucson, AZ). Changes in [Ca^2+^]_i_ were monitored in cells pretreated for 24 h with 10 nM BK and then stimulated by SDF-1 (3 or 30 ng/mL) or Bz-ATP (100 μM) compared to control experiments without BK pretreatment. The ionophore 4-Br-A23187 (5 μM) and the chelating compound EGTA (10 mM) were used to determine maximal (Fmax) and minimal (Fmin) fluorescence values, respectively. [Ca^2+^]_*i*_ values were calculated from relative fluorescence values using the equation [Ca^2+^]_*i*_ = K_d_ (F – Fmin)/(Fmax – F), assuming a K_d_ of 450 nM for fluo-3 calcium binding (Lameu et al., 2010). Calculated concentrations are mean values of data from at least 30 individual-analyzed cells.

### Calcium measurements by microfluorimetry

Changes in [Ca^2+^]_*i*_ of neuroblastoma cell populations were determined by microfluorimetry using the FlexStation III (Molecular Devices Corp.). Cells were incubated for 60 min at 37°C with the FlexStation Calcium Assay Kit (Molecular Devices Corp.) containing 2.5 mM probenecid in a final volume of 200 ml per well. Fluorescence of samples was excited at 485 nm, and fluorescence emission was detected at 525 nm (Lameu et al., 2010).

### Pore formation

In order to analyze the effects of chronical exposure to BK on P2X7 receptor-induced pore formation, 5 × 10^5^ cells were pretreated for 24 h with the peptide at 10 nM concentration. Afterwards, cells were incubated for 2–3 min with Bz-ATP (100 μM) and ethidium bromide (20 μM). The plasma membrane permeability to ethidium bromide was analyzed by flow cytometry using the Attune flow cytometer (Thermofisher). Ethidium bromide emission fluorescence was recorded using a blue laser (488 nm) and an emission BP filter 574/26 nm (BL2 channel). The results were analyzed using the FlowJo v10.1r5 software (Ashland, OR, USA). Cells that had not been pretreated with BK were used as control.

### Cell viability assay

Cells were seeded in 96 well plates (10^4^ cells/well) at 37°C in 5% CO_2_. After 24 h of culture, cells were kept for another 24 h in medium supplemented with 0.2% BSA in the absence or presence of BK (10 nM), ATP (1 μM) and Bz-ATP (100 μM) or combination of BK plus ATP or Bz-ATP. 10 μL of MTT [(3-(4,5-Dimethylthiazol-2-yl)-2,5-diphenyltetrazolium bromide)] (10 mg/mL) was added to each well and incubated at 37°C for 4 h. The medium was removed and 100 μL DMSO was added and incubated for 1 h at room temperature. The absorbance was measured at 600 nm using FlexStation III (Molecular Devices Corp.). All experiments were performed in triplicates with three different passage numbers of the cell.

### Cell proliferation

Cells were plated in culture flasks at an initial density of 10^4^ cells/cm^2^ in presence or absence of BK (10, 30, or 1,000 nM). Cells were counted after 24, 48, and 72 h by flow cytometry (LSRII flow cytometer, Becton & Dickinson).

### Transplantation of human neuroblastoma cells into nude/nude mice and short-term dissemination assay

To evaluate the *in vivo* behavior metastatic of neuroblastoma to the BM, lung and liver was injected 2 × 10^6^ cells into the tail vein of nude/nude control mice or mice i.p. injected with 50 mg/kg Brilliant Blue G (BBG), a P2X7 receptor antagonist (Ryu et al., 2011). The dissemination assays protocols were previously approved by the Ethics' committee of the University of São Paulo (CEUA 13/2017). Animals were sacrificed 48 h after injection of neuroblastoma cells, and tumor cells levels in bone marrow, lungs and liver were assessed. Relative quantities of human cells present in the murine organs were determined by real-time PCR assessment of human α-satellite DNA from these tissues as measure of the degree of chimerism, as described elsewhere (Jankowski et al., 2003). Primer sequences are listed in Table 1.

### Tumor generation and long-term dissemination assay

For tumor induction, 2 × 10^6^ CHP-100 cells pretreated for 24 h with 10 nM BK were inoculated into subcutaneous fat of the right limb. Animals were treated i.p. with 50 mg/kg BBG every 2 days, while the control group received 0.9% sterile saline solution. At the time of injection, mice were examined to assess general health conditions and to evaluate tumor growth. The major tumor mass diameter was measured with a manual caliper. On day 28 after inoculum, mice were sacrificed, tumor weight was measured, and relative human neuroblastoma cell levels in murine bone marrow were determined by qPCR as described above.

## Results

### Presence of the BK precursors in irradiated bone marrow

For providing proof for our hypothesis that radiotherapy provides a pro-metastatic microenvironment in BM, involving BK release, for seeding of tumor cells that survived treatment, alterations of kininogen expression and BK levels were determined in the irradiated BM.

**Figure 1:**
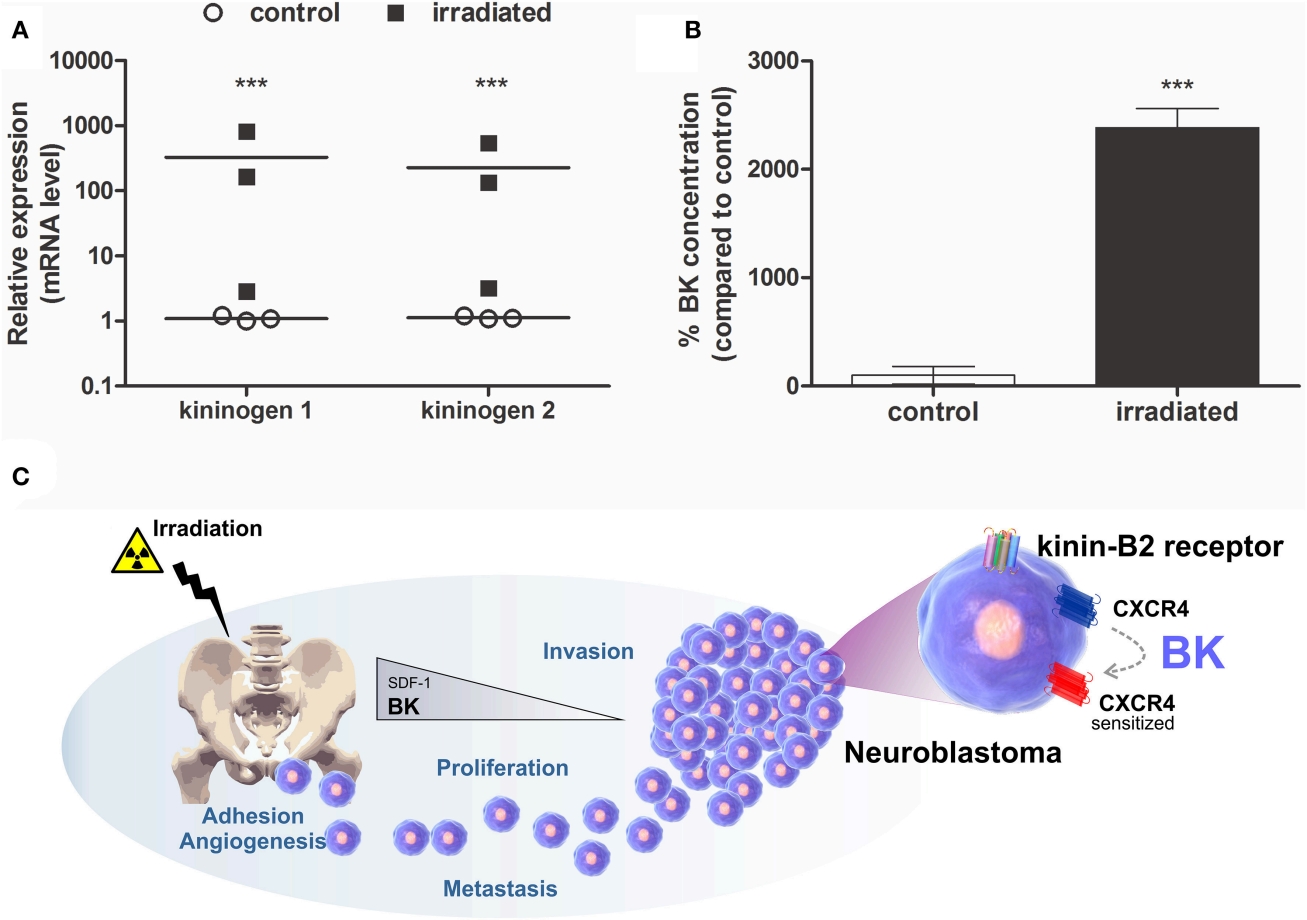
Expression of Bradykinin in Bone Marrow. BK concentration and it precursor RNA level were determined in murine bone marrow lysates prepared from the animal 24 h after irradiation (1,000 cGy). **(A)** mRNA levels of kininogen 1 and kininogen 2. Measurements were performed for samples from three independent isolations. Each analysis was performed in triplicates. **(B)** BK levels in bone marrow of irradiated mice compared to non-irradiated mice (control). The data are shown as mean of six independent experiments and plotted as percentages compared to the control (100%). ^***^p <0.001 compared to control. **(C)** Mechanistic illustration of BK-promoted neuroblastoma metastasis. BK levels increase in the bone marrow of animals that received radiation, a situation mimicking radiotherapy during cancer treatment. In this case, the levels of SDF-1, the main chemo-attractor of hematopoietic stem cells to the bone marrow, is reduced. However, BK as priming agent sensitizes the SDF-1 receptor (CXCR4), also expressed by neuroblastoma cells, induces expression of metalloproteinase (MMP-2 and/or MMP-9) important for tumor invasion and VEGF, a key protein in the mechanism of angiogenesis, and enhances adhesion and proliferation of neuroblastoma cells.

BK precursor (kininogens-1 and 2) mRNA transcription levels were detected by qPCR in much higher concentrations in BM cells of γ-irradiated mice when compared to non-irradiated animals (Figure 1A). Furthermore, BK levels were significantly augmented in BM of irradiated animals compared to control animals, as determined by ELISA (Figure 1B). Consequently, BK released by damaged BM contributes to a favorable microenvironment for metastasis formation. Based on that, we postulate that BK, secreted by the BM, enhances adhesion, invasion, angiogenesis and sensitizes CXCR4 to promote metastasis of neuroblastoma cells to the BM (Figure 1C).

### Bradykinin induces neuroblastoma cells to transform into pro-metastatic phenotypes

**Figure 2:**
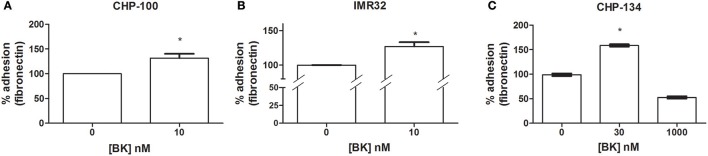
Effects of Bradykinin on adhesion. Adhesion rates determined as binding rates to fibronectin-coated dishes were measured following 24 h of culturing of neuroblastoma cells in the absence or presence of BK. **(A)** CHP-100 and **(B)** IMR-32 treated with 10 nM BK for 24 h; and **(C)** CHP-134 treated with 30 or 1,000 nM BK. Control experiments were performed in the absence of BK (adhesion rates normalized to 100%). ^*^p < 0.05 compared to untreated cells.

BK-mediated priming effects on the promotion of pro-metastatic features of tumor cells. Further, BK induced an increase of adhesion at a 10 nM dose of the peptide for CHP-100 (Figure 2A) and IMR-32 cells (Figure 2B), and at a 30 nM dose for CHP-134 cells (Figure 2C), respectively. The used BK concentrations had been effective in chemoattraction assays.

**Figure 3:**
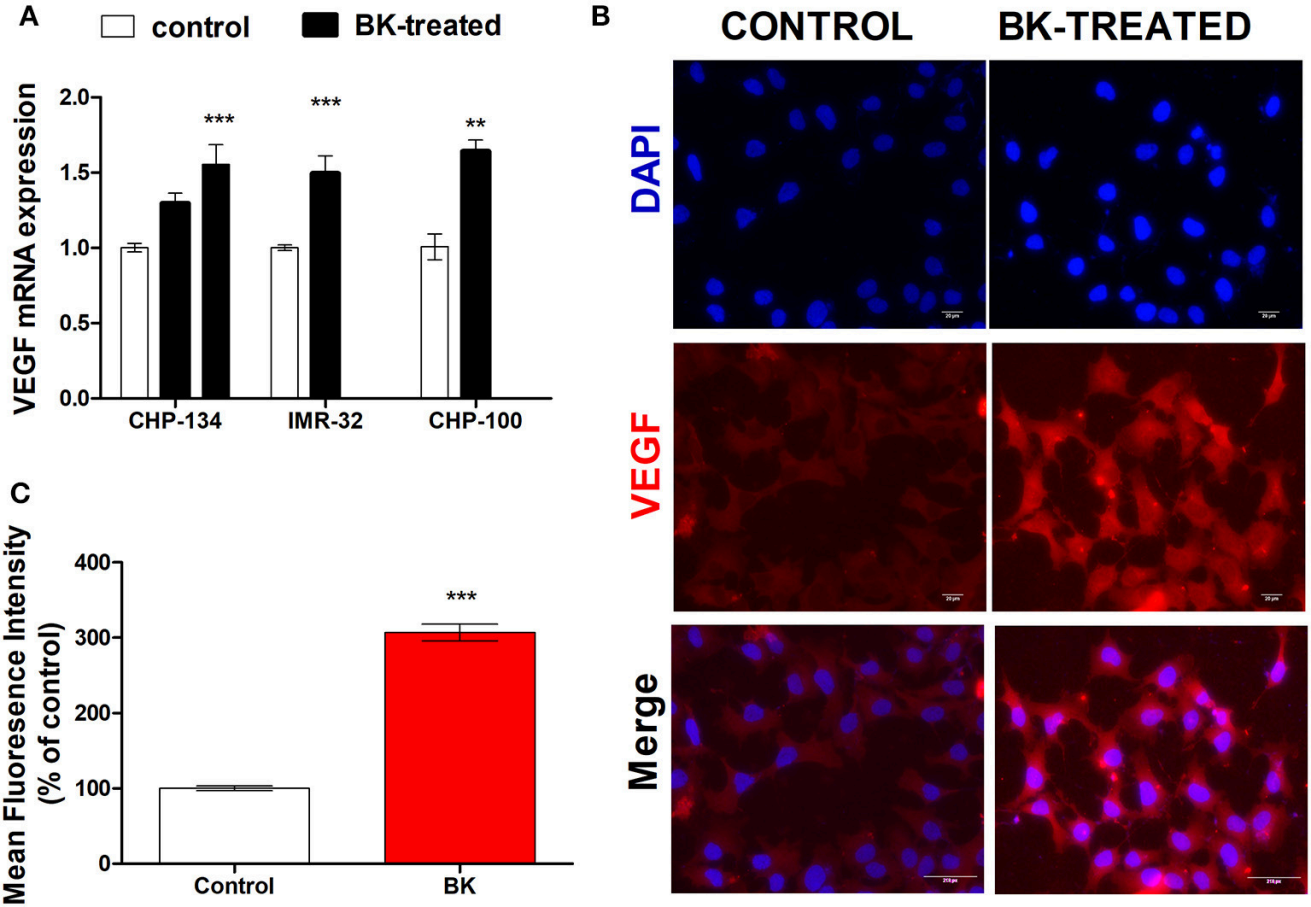
VEGF expression in neuroblastoma cell lines. CHP-100 and IMR-32 cells were treated with 10 nM BK and CHP-134 with 30 (left black bar) or 1,000 nM (right black bar). **(A)** Analysis of mRNA expression of VEGF was performed for three cell lines by real time PCR, using the method of relative quantification. The experiment was done three times. **(B)** Immunostaining for VEGF protein expression in BK-treated CHP-100 cells. Scale bar, 20 μm. **(C)** Intensities of fluorescence were analyzed using ImageJ software: Cell Magic Wand Tool. The data represent mean values of three independent experiments. ^**^p < 0.01 and ^***^p < 0.001 compared to respective controls.

Moreover, increased expression rates of VEGF in BK-treated neuroblastoma cells further support the hypothesis that this kinin is a pro-metastatic factor (Figures 3A–C). BK induced a significant increase in VEGF gene expression in CHP-134, IMR-32, and CHP-100 cells (Figure 3A) as well as of VEGF protein expression (Figure 3B). BK-treatment elevated VEGF protein density approximately three-fold, when compared to control experiments with CHP-100 cells (Figure 3C).

**Figure 4:**
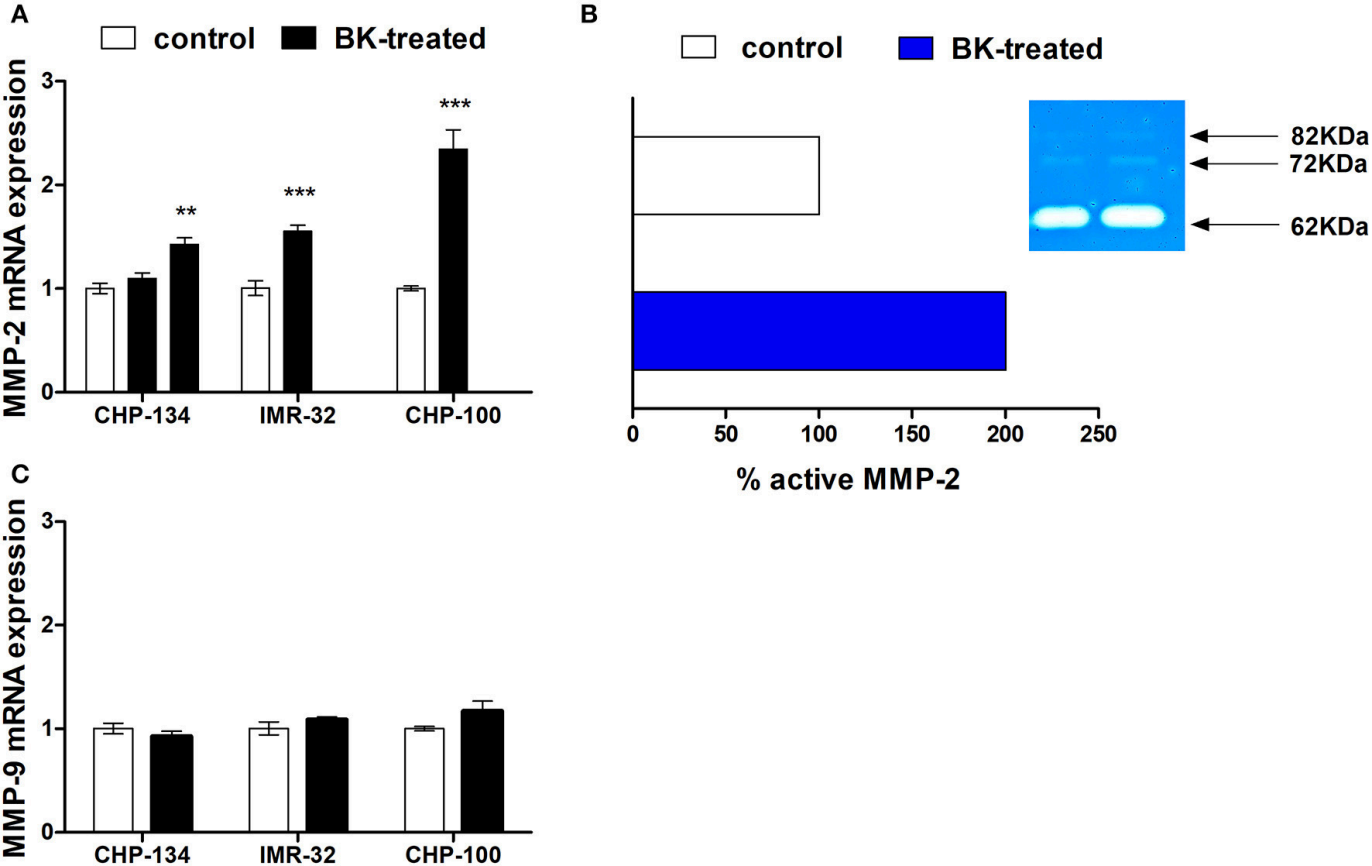
Metalloproteinase expression and activity in neuroblastoma. **(A)** CHP-100 and IMR-32 cells were treated with 10 nM BK and CHP-134 with 30 (left black bar) or 1,000 nM (right black bar) and analysis of mRNA expression of MMP-2 was performed for three cell lines by real time PCR, using the method of relative quantification. The experiment was done three times. **(B)** Graphic plot of the mean relative intensity regrading active form of MMP-2 (62 KDa) of the bands obtained in the zymography assay and representative gelatin zymogram: the active latent form of MMP-9 (82KDa), latent (72 KDa) and active forms (62 KDa) of MMP-2 were detected. **(C)** Analysis of mRNA expression of MMP-9 was performed by real time PCR. In this experiment CHP-134 was treated only with 1,000 nM BK. The data represent mean values of three independent experiments. ^**^p <0.01 and ^***^p <0.001 compared to respective controls.

Matrix metalloproteinases (MMPs), especially MMP-2 and MMP-9, are responsible for the degradation of components of the basement membrane and extracellular matrix, thereby promoting tumor invasion (Aznavoorian et al., 1993; Curran and Murray, 2000; Rundhaug, 2005). BK treatment enhanced MMP-2 gene expression in CHP-134, IMR-32 and CHP-100 cell lines (Figure 4A). In addition to the gelatinolytic activity of stimulated MMP-2 (62 kDa), its latent MMP-2 form (72 kDa) and the active form of MMP-9 (82 kDa) in CHP-100 cells were detected by zymography assays (Figure 4B). Only MMP-2 activity was augmented upon treatment with BK, while activity and expression levels of MMP-9 were unchanged following BK treatment (Figures 4B,C).

**Figure 5:**
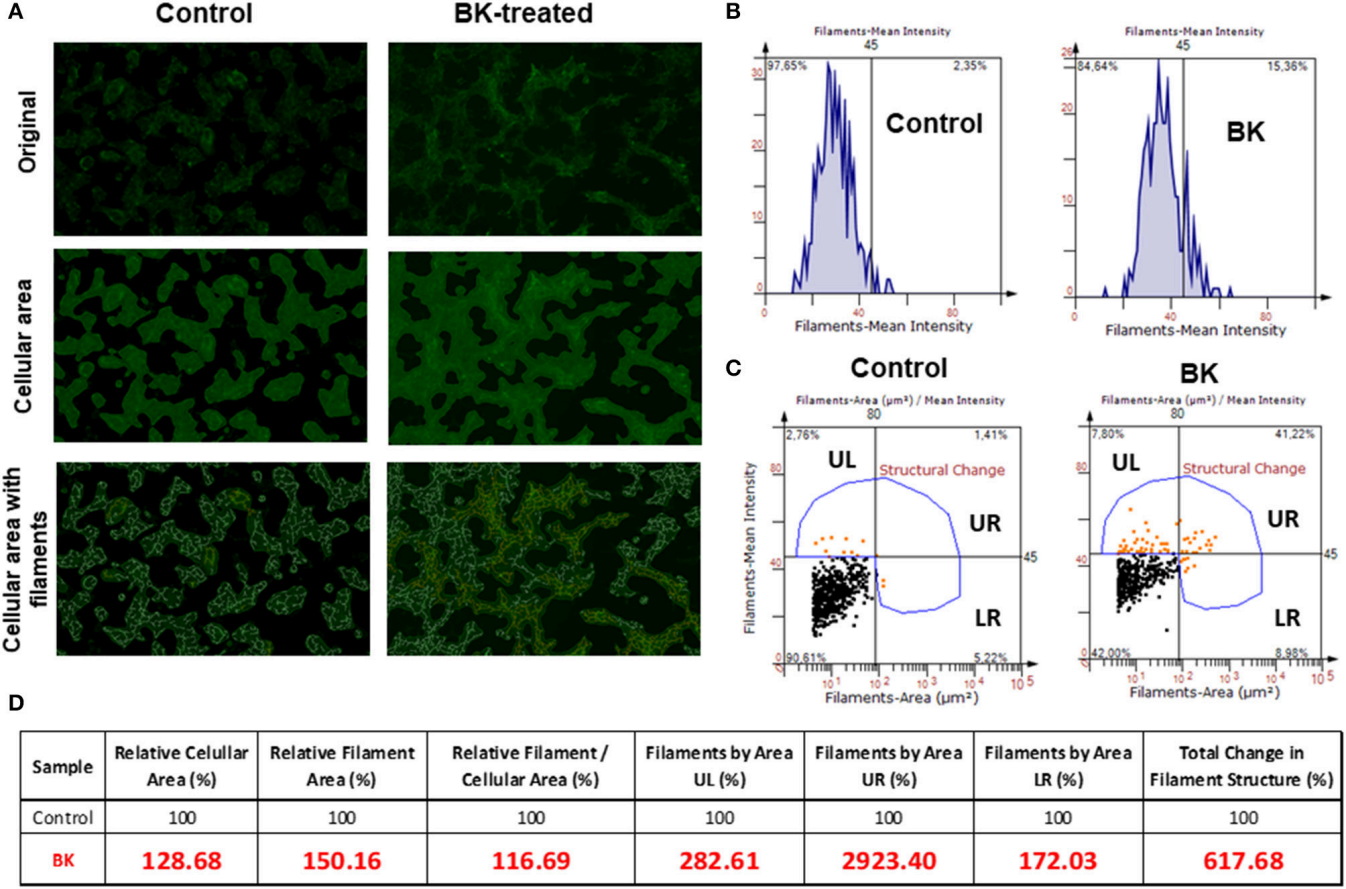
Rearrangements of the actin cytoskeleton in neuroblastoma cells induced by Bradykinin. **(A)** Fluorescence microscopy images are representative of actin filament staining with Alexa Fluor 488 phalloidin (original); images with cell area demarcation and with outstanding filaments. **(B)** Representative histograms of fluorescence intensity of actin filaments of control and BK-treated CHP-100 cells. **(C)** Quantitative analysis of change in filament structure (fluorescence intensity X cellular area) in neuroblastoma cells treated with 10 nM BK compared to control using Strata Quest software. The quadrants were divided as UL (small filaments that are intensely stained), UR (large filaments that are intensely stained), and LR (large filaments that are weakly stained). **(D)** Table of relative values analyzed parameters to identify changes in filaments structure. The experiment was repeated three times.

Next, Alexa phalloidin immunofluorescence staining for the actin cytoskeleton, revealed notable morphological differences between BK-treated and untreated CHP-100 cells (Figure 5A), although overall staining intensity did not show any significant difference (Figure 5B). For quantitative analysis of changes structural of actin filaments (F-actin), cellular areas were identified, followed by measurement of filament areas and staining intensities using Strata Quest Software (TissueGnostics). In the control sample, there were much less filaments, but also cellular staining areas were smaller than in BK-treated samples. Hence, when the filament area was normalized to the cellular area, the total difference in the number of filaments was not as expressive. The real difference, however, comes from the structural differences, as seen in the 3 quadrants UL (small filaments that are intensely stained), UR (large filaments that are intensely stained), and LR (large filaments that are weakly stained) (Figure 5C). In the absence of BK, neuroblastoma cells displayed smaller cellular areas with short F-actin filaments, but in its presence, cells revealed larger cellular area with longer filaments compared to control cells. Short branched filaments were more strikingly stained, reflecting BK-induced structural changes of F-actin (Figure 5D).

### Influence of bradykinin and ATP on chemotaxis of neuroblastoma cells

**Figure 6:**
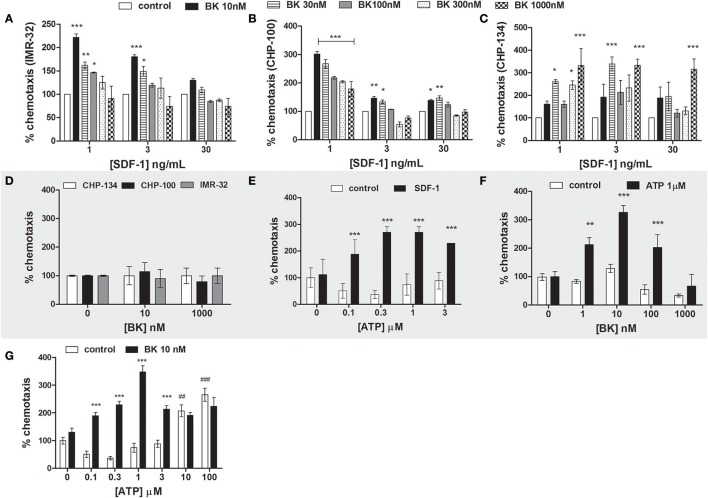
Neuroblastoma chemotaxis. **(A–C)** Priming effect of increasing BK concentrations on neuroblastoma cell chemotaxis in response to stimulation with 1, 3, or 30 ng/ml SDF-1 (**A**: IMR-32; **B**: CHP-100 and **C**: CHP-134). **(D)** Chemotaxis of neuroblastoma cells (CHP-134, CHP-100, and IMR-32 cell lines) in response to BK treatment. Cells were seeded into the upper chamber of the transwell insert. The bottom part was filled with 0, 10, or 1,000 nM of BK in 0.2% BSA. **(E)** Priming effect of increasing ATP concentrations on CHP-100 neuroblastoma cell chemotaxis in response to stimulation with 3 ng/ml SDF-1. **(F)** Priming effects of 1 μM ATP on CHP-100 neuroblastoma cell chemotaxis in response to stimulation with increasing concentrations of BK. **(G)** Priming effect of 10 nM BK on t CHP-100 neuroblastoma cell chemotaxis in response to ATP stimulation. The experiments were done at least three times ^*^p < 0.05; ^**^p < 0.01, and ^***^p < 0.001 compared to the corresponding dose in the control group. ##*p* < 0.01 and ###*p* < 0.001 compared to control without ATP.

We show here that BK treatment increased responsiveness of neuroblastoma cells to physiological SDF-1 concentrations. Various BK concentrations (10, 30, 100, 300, and 1,000 nM) were screened for determining the optimal concentration for its priming effect on three neuroblastoma lines (CHP-134, IMR-32, and CHP-100) in response to 1, 3 and 30 ng/mL SDF-1. The concentration of 10 nM BK significantly increased the migration of IMR-32 and CHP-100 cells in the presence of low doses of SDF-1, while this BK concentration did not result in any significant alteration of migration in the presence of high doses of SDF-1. Maximal chemotaxis effects upon administration of 1 ng/mL SDF-1, visible as 250 and 300% increases in migration rates compared to control experiments, were observed with IMR-32 and CHP-100 lines, respectively, when cells had been treated for 24 h with 10 nM BK (Figures 6A,B). CHP-134 cells revealed biphasic effects for BK priming. Cells pretreated for 24 h with 30 or 1,000 nM BK revealed highest priming rates, with 350% of control values, at all assayed doses of SDF-1 (Figure 6C). However, BK alone did not act as chemoattractant for neuroblastoma cells, neither at low (10 nM) nor at high doses (1,000 nM) (Figure 6D).

Increasing concentrations of ATP were applied together with 3 ng/mL SDF-1 in order to determine the priming effect of extracellular nucleotides in response to SDF-1. Such as BK, ATP exerted pronounced priming effects at all assayed concentrations (Figure 6E).

Of interest, 1 μM ATP primed neuroblastoma cell migration toward the BK in relation to different BK concentrations, increasing chemotaxis of neuroblastoma cells by 200–300%, showing maximal effects in response to 10 nM BK stimulation (Figure 6F). ATP alone was chemoattractant for neuroblastoma cells if employed at supra-physiological doses (10 and 100 μM), as extracellular ATP is basically undetectable in healthy tissues (Junger, 2008). In contrast, cells primed with 10 nM BK became responsive to up to 3 μM physiological concentrations of ATP (Junger, 2008), while such priming effect was not observed at high doses of ATP (10 and 100 μM) (Figure 6G), indicating an interplay of BK and ATP signaling in cell chemotaxis.

### CXCR4 are expressed in neuroblastoma cell lines

**Figure 7:**
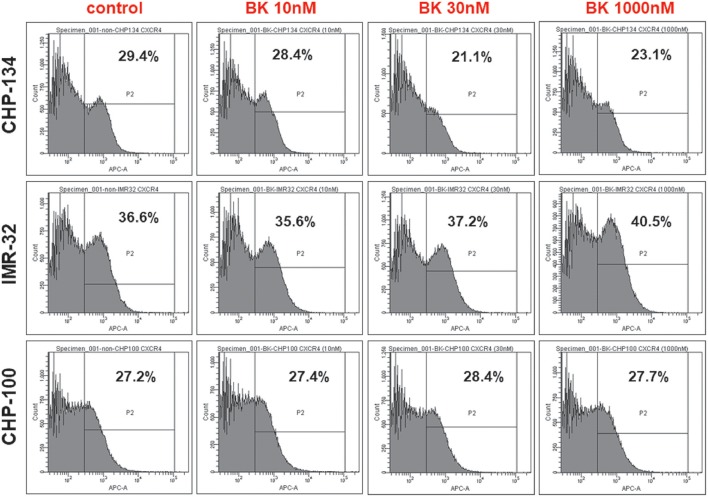
Neuroblastoma cell lines express (C-X-C chemokine receptor type 4). Flow Cytometry Analysis of CXCR4^+^ cells in CHP-134, IMR-32, and CHP-100 cell lines under increasing BK concentrations. Data representative of three independent assays.

CXCR4, the receptor for the chemokine stromal-derived factor 1 (SDF-1), mediates essential processes for tumor progression, such as metastasis. In order to investigate, whether CXCR4 expression was enhanced at the neuroblastoma cell surface in response to exogenously added BK explaining the increased responsiveness of these cells to SDF-1 in the chemotaxis assay, flow cytometry analysis was undertaken with CHP-134, IMR-32, and CHP-100 cell lines. As shown in Figure 7, all tested cells lines expressed CXCR4, while cell surface expression levels of this chemokine receptor remained unchanged following 24 h treatment with increasing concentrations of BK.

### Bradykinin regulates expression rates of purinergic receptors

**Figure 8:**
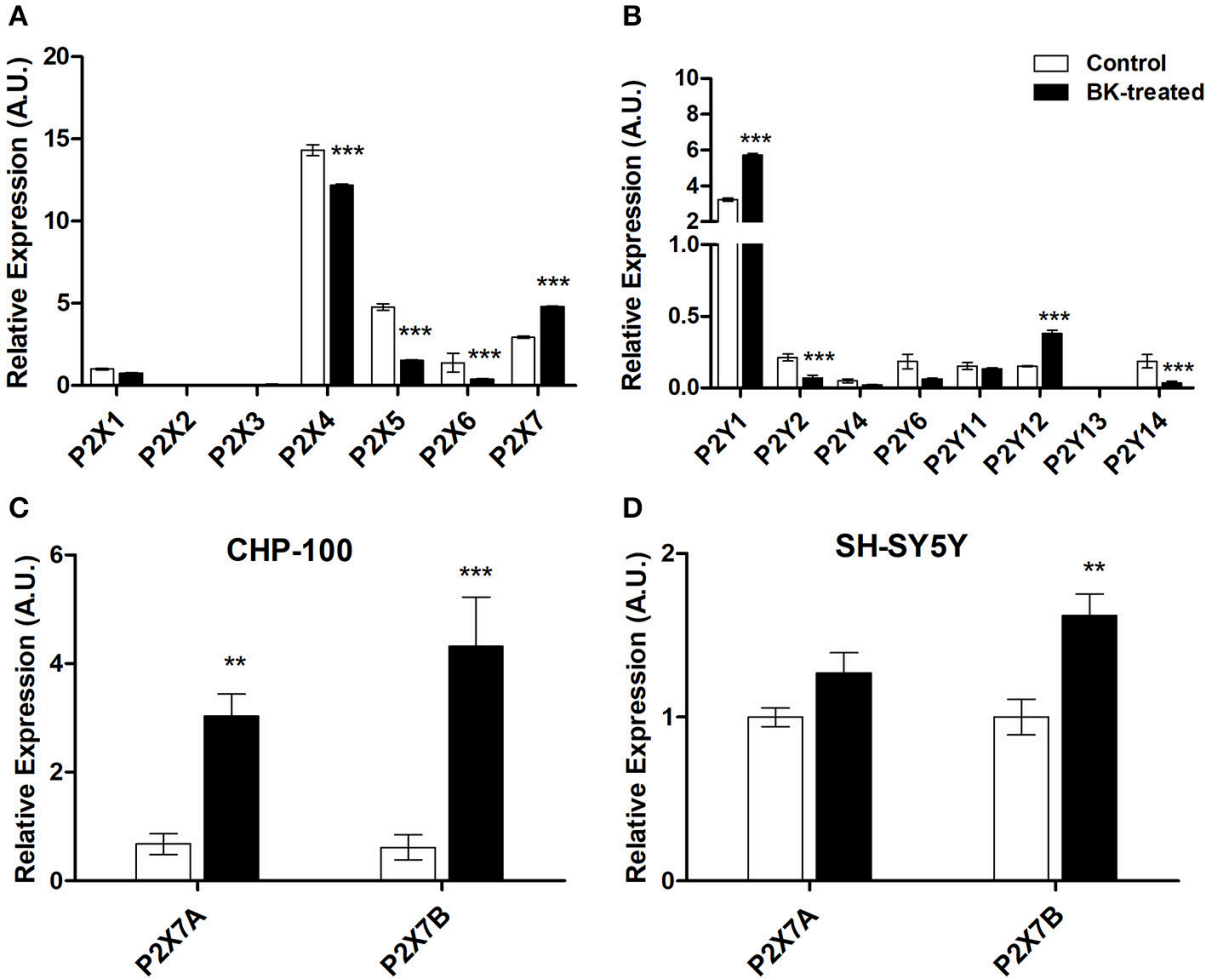
P2 Purinergic receptors expressed by neuroblastoma cells under BK treatment. Differential gene expression of P2 purinergic receptors was assessed by real-time PCR in CHP-100 and SH-SY5Y cells maintained in BSA 0.2% for 24 h with or without 10 or 30 nM BK, respectively, and normalized using glyceraldehyde 3-phosphate dehydrogenase (GAPDH) mRNA levels. mRNA expression changes of **(A)** P2X receptor subtypes; **(B)** P2Y receptor subtypes; **(C)** P2X7 isoforms A and B in CHP-100 cells; **(D)** P2X7 isoforms A and B in SH-SY5Y cells. The obtained data were compared with mRNA levels of non-treated CHP-100 cells (control) expressed as arbitrary units (A.U.).^**^p < 0.01 and ^***^p < 0.001.

Interrelationships between purinergic and kinin signaling were observed for *in vitro* chemotaxis of neuroblastoma cells, such as in our earlier work in the context of neural migration and differentiation (Trujillo et al., 2012). In our present work, exposure of neuroblastoma cells for 24 h to BK decreased P2X4, P2X5, P2X6, and P2Y2 receptor subtype expression, while expression rates of P2X7, P2Y1, and P2Y12 subtypes in CHP-100 cells were enhanced (Figures 8A,B). Visibly, exposure of neuroblastoma cells to BK also upregulated both P2X7 receptor A and B splice variant expression in CHP-100 (Figure 8C) and only P2X7B receptor variant expression in SH-SY5Y cells (Figure 8D). It is noteworthy that both cell lines originate from the metastatic BM site.

### Sensitization of CXCR4 and P2X7 receptors by BK

**Figure 9:**
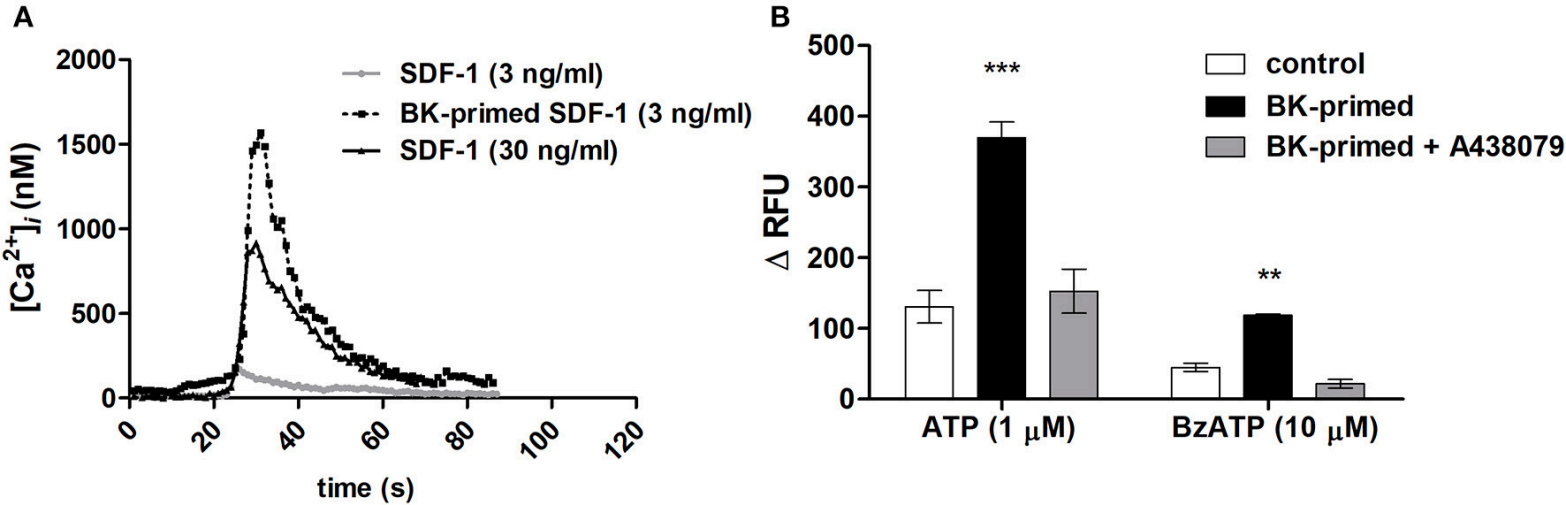
Elevations of [Ca^2+^]_*i*_ levels in neuroblastomas cells after stimulation with SDF-1, ATP or Bz-ATP. Measurements were performed with cells primed for 24 h with 10 nM BK and compared to those obtained with untreated control cells. **(A)** Representative traces of [Ca^2+^]_*i*_ transients upon SDF-1 (3 or 30 ng/ml) application determined by calcium imaging. **(B)** For microfluorimetry-based [Ca^2+^]_*i*_ measurements, 1 μM ATP or 10 μM Bz-ATP were used to stimulate control cells or cells primed for 24 h with 10 nM BK. A438079 (5 μM) was used to determine participation of the P2X7 receptor subtype in purinergic receptor responses. Data are presented as the difference between maximal and minimal relative fluorescence units (ΔRFU). ^**^p < 0.01 and ^***^p < 0.001.

The specific effects of the SDF-1 are mediated by G-protein–linked seven-trans-membrane domain receptors. A transient dose-dependent increase in ([Ca^2+^]_*i*_) occurs as an essential component of the signal transduction cascade activated upon binding of a chemokine to its receptor (Roy et al., 2017). Neuroblastoma cells responded with transient elevations of [Ca^2+^]_*i*_, when stimulated with 30 ng/mL SDF-1. Exposure for 24 h to 10 nM BK sensitized neuroblastoma cells to low doses of SDF-1 (3 ng/mL) (Figure 9A), suggesting that BK increases the sensitivity of CXCR4 responsiveness to SDF-1 facilitating its activation. Moreover, cells treated with BK showed augmented responses to P2X7 receptor agonist Bz-ATP and ATP stimulation. Effects induced by both ATP and Bz-ATP were inhibited by A438079, a selective P2X7 receptor antagonist (Figure 9B).

### Bradykinin induces neuroblastoma cell proliferation

**Figure 10:**
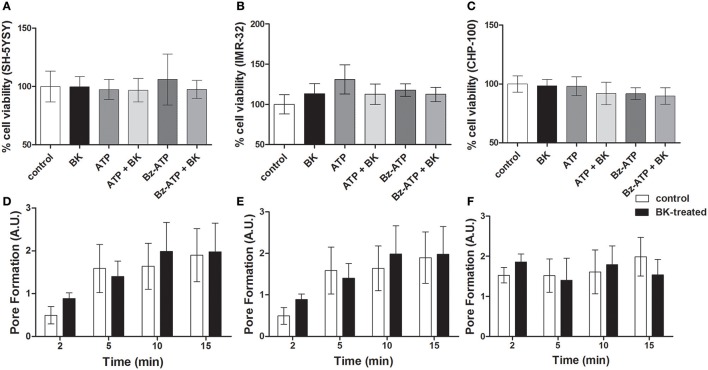
Cell viability assays and P2X7 receptor-induced pore formation in BK-treated neuroblastoma cell lines. Neuroblastoma cell lines **(A)** SH-5YSY; **(B)** IMR-32, and **(C)** CHP-100 exposed to different treatments for 24 h were screened for induction of cell death. Control measurements (without treatment) were normalized to 100%. Pore formation was determined as membrane permeability for ethidium bromide (20 μM) was measured by flow cytometry in **(D)** SH-5Y5Y, **(E)** IMR-32, and **(F)** CHP-100 cells. The cells had been pre-treated with BK (10 nM) for 24 h and were then incubated for 2, 5, 10, and 15 min with Bz-ATP (100 μM). Cells treated only with Bz-ATP and ethidium bromide served as control. Data are presented as mean values ± SD of three independent experiments.

**Figure 11:**
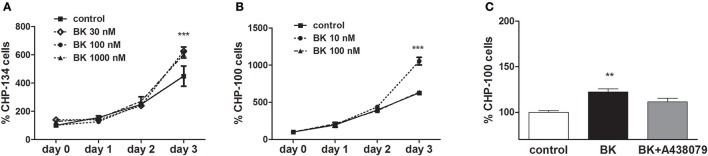
Effects of bradykinin on proliferation. Proliferation kinetics were assessed for 3 days following BK addition: **(A)** CHP-134 in the absence (control) or presence of 30, 100, or 1,000 nM BK. **(B)** CHP-100 in the absence (control) or presence of 10 or 100 nM BK. **(C)** Proliferation rate on day 3: CHP-100 in the absence (control), presence of 10 nM BK or presence of BK 10 nM and 5 μM A438079, a selective P2X7 receptor antagonist. Data represent mean values ± S.D. of four independent experiments. ^**^p < 0.01; ^***^p < 0.001 compared to control.

P2X7 receptor activation in ACN neuroblastoma cells does not induce cell death, while driving proliferation (Raffaghello et al., 2006). Accordingly, in our hands, exposure to BK; BK + Bz-ATP or BK + ATP did not decrease cellular viability in at least three of the investigated neuroblastoma cell lines (Figures 10A–C). Similarly, P2X7 receptor-induced pore formation did not augment, when the same neuroblastoma cell lines had been pretreated with BK (Figures 10D–F). As expected, BK at 10–1,000 nM concentrations promoted proliferation of both, CHP-134 (Figure 11A) and CHP-100 cells (Figure 11B). Of interest, BK-promoted effects on the proliferation rate was significantly decreased in the presence of the P2X7 receptor antagonist A438079 (Figure 11C), suggesting that kininergic-purinergic signaling cross talking is required for BK-dependent cancer cell proliferation.

### Participation of BK and P2X7 receptors in site-directed spreading of neuroblastoma cells to organs expressing SDF-1

**Figure 12:**
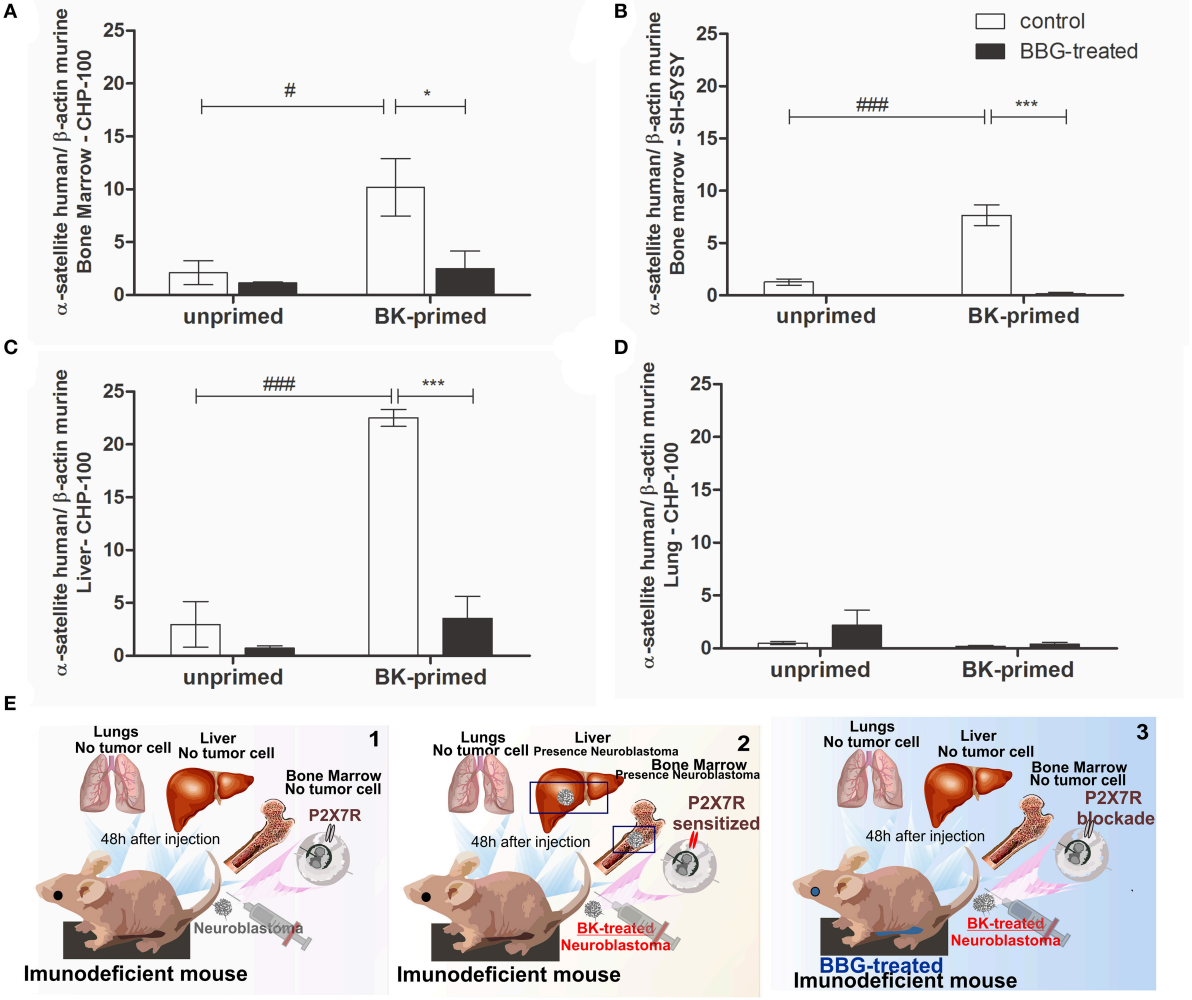
Priming effects of bradykinin and P2X7 receptor antagonism on cancer dissemination. Unprimed control group: about 2 × 10^6^ cells were injected i.v. into nude/nude mice; Unprimed BBG-treated group: animals were also treated i.p. with 1 mg/kg Brilliant Blue G (BBG); BK-primed control group: approximately 2 × 10^6^ cells treated with 10 nM BK for 24 h were injected i.v. into nude/nude mice; BK-primed BBG-treated group: treated i.p. with BBG (50 mg/kg). Following 48 h of injection of BK-pretreated neuroblastoma cells, liver and lungs were extracted, and cavities of the femur, tibia and humerus were washed for isolation of bone marrow cells. Metastasis rates of **(A)** CHP-100 and **(B)** SH-SY5Y cells in murine bone marrow, CHP-100 cells in **(C)** liver and **(D)** lungs were measured as differences in levels of α-satellite DNA amplified in human extracts isolated from cells derived from bone marrow, using real-time PCR. Data are mean values of 4 independent experiments per group. The results are shown as mean values ± SEM. ^#^*p* < 0.05 and ^###^*p* < 0.001 compared to unprimed control experiments; ^*^p < 0.05 and ^***^p < 0.001 compared to BK-primed control. **(E)** Schematic illustration of *in vivo* assays: 1, animals injected with neuroblastomas cells; 2, animals injected with BK-treated neuroblastoma cells and 3, animals treated with BBG following injection of BK-treated neuroblastoma cells.

Finally, the effects of BK and of P2X7 receptor antagonism on metastatic dissemination of neuroblastoma cells to BM, liver and lungs were evaluated in short and long-term transplanted animal models. We tested, whether the treatment of neuroblastoma cells with BK and inhibition of P2X7 receptor in nude mice affected the metastatic spread (seeding efficiency) of these cells to tissues expressing SDF-1 in a short-term metastatic model (Figure 12). We found that BK significantly increased the seeding efficiency of neuroblastoma cells in liver and BM and that this effect was significantly decreased after treatment of animals with the P2X7 receptor antagonist Brilliant Blue-G (BBG) (Figure 12A–C). We noticed that the seeding efficiency to the BM increased about 8 and 10 times for CHP-100 and SH-SY5Y lines, respectively, when cells had been treated with BK *ex vivo* (Figures 12A,B) and 25 times more for CHP-100 cells to the liver (Figure 12C) after 48 h of i.v. injection of cells into immunodeficient nude mice. However, when animals had been i.p. treated with the P2X7 receptor antagonist BBG, neuroblastoma cells lack the ability to spread to these organs even when these had been *ex vivo* primed with BK (Figures 12A–C). In contrast, the spreading of neuroblastoma cells to the lungs showed no significant difference regardless if tumor cells had been primed with BK or treated with BBG (Figure 12D). A schematic illustration of these findings has been presented (Figure 12E).

**Figure 13:**
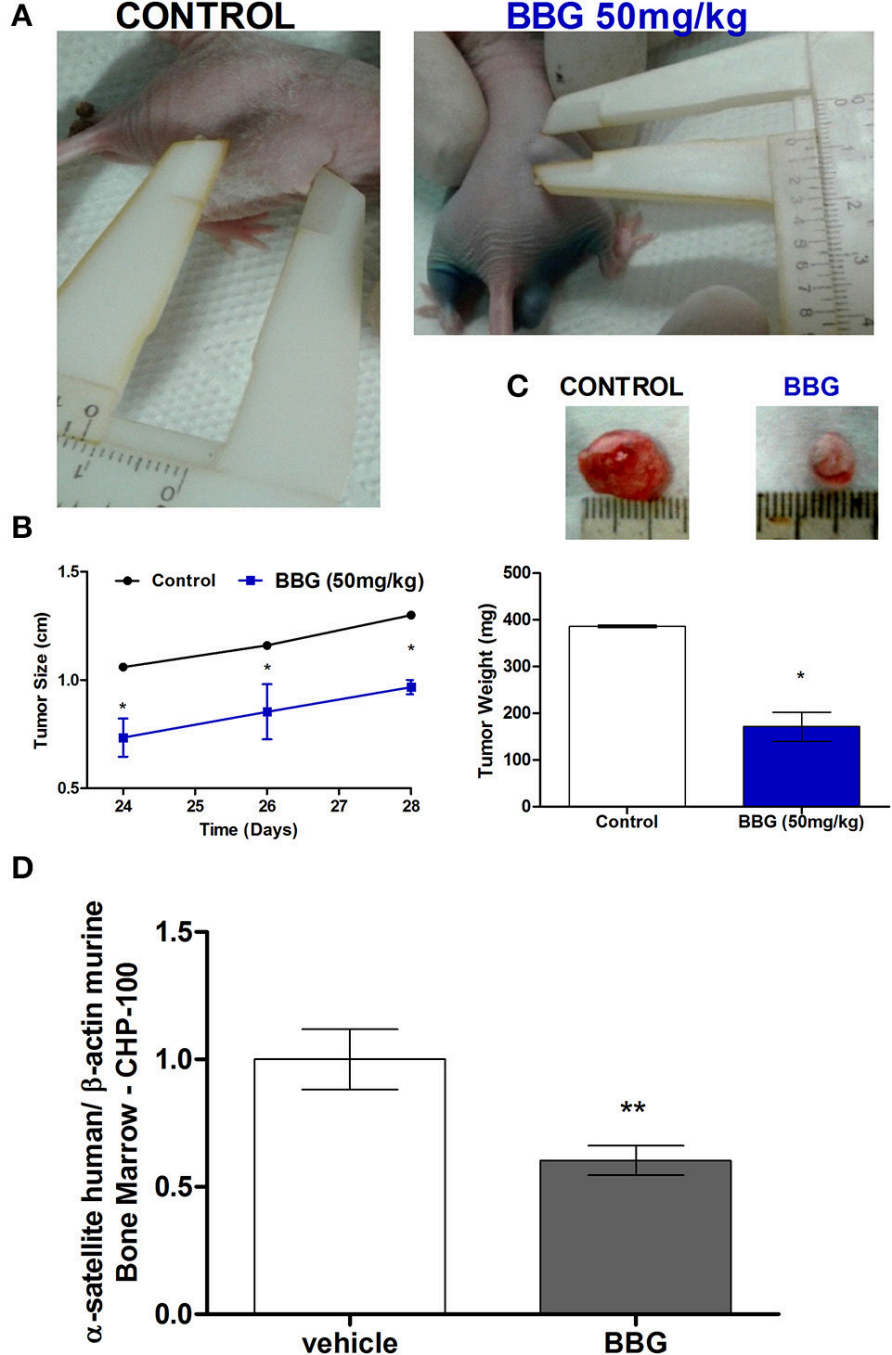
Long-term metastasis assay. Nude/nude mice were s.c. xenotransplanted with about 2 × 10^6^ cells, which had been pretreated with 10 nM BK for 24 before injection. These mice were divided in 2 groups: animals receiving i.p. vehicle or 50 mg/kg BBG every 48 h. **(A)** Representative photograph for demonstrating sizes of xenotransplanted tumor in nude/nude mice on day 24. (**B**) Comparison of tumor size over time of BBG-treated mice and control mice. **(C)** Representative tumor mass excised on day 28 post-inoculum and comparison of tumor weight between control animals and BBG-treated animals. **(D)** Human neuroblastoma cells (CHP-100) in murine bone marrow were relatively quantified by real-time PCR measuring differences in levels of α-satellite DNA amplified in human extracts isolated from cells derived from bone marrow. Data are mean values of 4 independent experiments per group. The results are shown as mean values ± SD. ^*^p < 0.05 and ^**^p < 0.01.

In order to assess interrelationship of kinin and purinergic systems in a long-term animal model, nude mice were subcutaneously inoculated with BK-treated CHP-100 cells, then the animals were received i.p. BBG or saline every 2 days. Mice receiving saline developed palpable tumors within 10 days after inoculation, while tumor masses in mice injected with BBG occurred later on day 15 (Figure 13A). Size and weight of tumors of saline-receiving animal was significantly larger than that of BBG-treated animals (Figures 13A–C). As expected, seeding efficiency to BM was significantly decreased with chronical treatment with BBG. This result corroborates with data of short-term model (Figure 13D).

## Discussion

The pivotal role of the (SDF-1)/CXCR4 axis in BM metastasis has been described for various tumor types, including neuroblastoma (Russell et al., 2004; Kucia et al., 2005b). However, blocking of the SDF-1-CXCR4 axis does not prevent metastasis of cancer cells to the BM and other tissues expressing SDF-1, suggesting the involvement of other pro-metastatic factors as chemoattractants for tumor cells (Wysoczynski et al., 2007). Based on well-known side effects of chemotherapy and radiotherapy, mainly due to the leaking of inflammatory molecules from damaged organs, we propose BK and extracellularly acting ATP as potential pro-metastatic factors for neuroblastoma cells.

Previously, we reported that extracellular nucleotides are released during radio- and chemotherapy by the BM (Schneider et al., 2015). In the present study, we show that the transcript levels of BK precursor as well peptide BK concentration are significantly increased in BM from irradiated mice. These data corroborate with our hypothesis that molecules related to inflammation, such as BK and ATP, are released in large amounts by damaged BM due to unwanted effects of radio- and chemo-therapy.

We have suggested that increased levels of BK and ATP point at a compensatory effect for metastasis of tumor cells to the BM, explaining why this tissue is the most common site for recurrence of several cancers (Coleman, 2001; Bacci et al., 2006), even when SDF-1 levels diminish after chemotherapy decreases due to an induced proteolytic environment (Ratajczak et al., 2013).

The pioneering discovery of the present study is the action of BK, as a pro-metastatic factor, as well as its relationship with the SDF-1/CXCR4 axis and purinergic signaling. BK-mediated effects on neuroblastoma progression include increasing cell adhesion, promoting angiogenesis, and rearrangement of the cytoskeleton, in addition to proliferation induction and enhancement of neuroblastoma invasion capacity due to sensitization of both CXCR4 and P2X7B receptors.

In view of that, increased adhesiveness of neuroblastoma cells is important for the establishment of a physical connection between metastatic cells and BM. In agreement we report herein that BK increased the adhesion ability of neuroblastoma cells to fibronectin. BK is known to provoke formation of focal adhesions in quiescent cells, as it promotes phosphorylation of paxillin and Focal adhesion kinase (FAK) (Leeb-Lundberg et al., 1994). Focal adhesions are specialized anchoring sites found in cultured cells, where extracellular domains of integrins bind to extracellular matrix proteins such as fibronectin (Burridge, 1988). This interaction results in the aggregation of integrins and their intracellular association with cytoskeletal proteins by domains that anchor filament bundles of polymerized actin (stress fibers) at these sites. FAK activation also is involved in regulating cell responses to environmental stimuli to influence tumor cell migration (Megison et al., 2013), through signal-mediated effects on actin organization. Here, we have shown that BK promoted structural changes of the actin cytoskeleton. This role of FAK in controlling actin-remodeling dynamics during tumor cell adhesion and motility is congruous with the observations that perturbed FAK expression and activity correlates with increased clinical progression into highly malignant and metastatic phenotypes (De Vicente et al., 2013).

For metastasis, invasion is an important and complex process involving several coordinated stages: egress of cancer cells from primary tumor, establishing new contacts with the environment, degradation and remodeling of the extracellular matrix and migration of tumor cells to new tissue locations (Rao, 2003). In this process, matrix metalloproteinases (MMP), especially MMP-2 and MMP-9, are crucial for extracellular matrix remodeling and, consequently, the tumor invasiveness. BK treatment resulted in increased expression and activity of MMP-2 in neuroblastoma cells. The proteolysis of the basement membrane and extracellular matrix is a prerequisite for the formation of new vessels that provide oxygen and nutrients as well as a novel routes for neoplastic cells to enter circulation and reach distant organs (Rundhaug, 2005; Folkman, 2007).

Among the multiple effects of BK on neuroblastoma cells, we have observed that BK induced VEGF expression. VEGF promotes angiogenesis with the growth of new blood vessels inside the tumor. These vessels nourish the tumors and at the same time provide and facilitate the emergence of metastasis. The increase in VEGF secretion could be a consequence of BK-dependent P2X7 receptor overexpression, as this purinergic receptor subtype was shown to cause a rise in tumor vascularization via VEGF secretion (Adinolfi et al., 2012; Amoroso et al., 2015).

We have observed that BK increases the sensitivity of CXCR4 in response to SDF-1 and facilitates its activation as shown by augmented amplitudes of [Ca^2+^]_*i*_ transients. The BK-induced responsiveness to SDF-1 could result from lipid rafts formation, a relevant mechanism for the neuroblastoma model (Palacios-Moreno et al., 2015; Ding and Zajac, 2016), since even after 24 h of contact with neuroblastoma lines, BK at various concentrations (10, 30, 100, 300, and 1,000 nM) did not alter CXCR4 expression levels, as evaluated by flow cytometry. However, we demonstrated that BK-treated neuroblastoma cells increased expression levels of P2Y1 and P2X7 receptors. These purinergic receptor subtypes are directly related to the metastasis of cancer cells (Shafat et al., 2006; Roger and Pelegrin, 2011).

Although BK or ATP alone did not exert any chemotactic effects on neuroblastoma cell lines, we demonstrate here their effects on inducing striking chemotaxis by increasing the response of those cells to low physiological-relevant doses of SDF-1, which resemble those encountered in endogenous physiological conditions (Junger, 2008).

We have paid special attention to the interplay between P2X7 receptor and BK actions, since several recent reports have correlated P2X7 receptor activity with carcinogenesis and, interestingly, BK-promoted effects were counteracted by pharmacological blockade of P2X7 receptor activity, both *in vitro* and *in vivo*. Upon BK treatment, sensibility toward the P2X7 agonist was increased, and this effect was reversed following application of a selective P2X7 receptor antagonist. The influence of BK on P2X7 receptor activity may explain its role and mechanism in promoting tumor cell dissemination, as different studies point at the P2X7 receptor as an important promoter of invasiveness (Jelassi et al., 2011).

Moreover, BK treatment increased the expression levels of the only two known active human isoforms of the P2X7 receptor: P2X7A and P2X7B. Although little is known about P2X7B isoform expression in physio-pathological conditions, it is clear that this truncated version of the P2X7 receptor retains the growth promoting activity of the full length P2X7A protein while losing the pro-apoptotic function (Adinolfi et al., 2010; Giuliani et al., 2014). Therefore, it is tempting to speculate that, the expression of the P2X7B isoform will be more advantageous for tumor growth as compared to the P2X7A one. This hypothesis is further supported by recent data of Giuliani et al. showing that, in osteosarcoma, P2X7B isoform overexpression associates to increased tumor cell proliferation as compared to the P2X7A isoform (Giuliani et al., 2014).

Accordingly, we have gathered evidence that BK treatment of neuroblastoma cells favors the upregulation of P2X7B vs. the P2X7A isoform. BK prompts cell proliferation; however, it does not decrease cell viability nor increases P2X7 receptor pore activity associated to the P2X7A isoform. Furthermore, BK-induced proliferation was significantly decreased by P2X7 receptor antagonism, suggesting that BK could upregulate P2X7 receptor-dependent signaling pathways leading to neuroblastoma growth (Amoroso et al., 2015). Moreover, BK-induced P2X7B isotype expression would allow neuroblastoma cells to exploit the high extracellular ATP concentration in the BM as a growth and seeding stimulus, rather than as a death signal.

Finally, we show here that BK promotes seeding of neuroblastoma cells in specific organs, being in line with results obtained *in vitro*. Human neuroblastoma cells were not detected in the lungs indicating preference of metastatic cancer cell migration to the BM and liver. This emphasizes the Paget's “seed and soil” hypothesis describing the interaction between the tumor cell and its environment in order for metastasis to occur (Paget, 1889). Furthermore, an interrelationship between purinergic and kinin systems was confirmed *in vivo*, where spreading of human neuroblastoma cells treated with BK to BM and liver was abolished or drastically reduced in BBG-treated nude mice compared to animals which had received a control solution. These data provide evidence for a novel mechanism of crosstalk between P2X7 and kinin signaling in the metastatic process.

The effect of P2X7 receptor blockade on BK-induced seeding to BM was also observed in a long-term animal model. The chronic treatment of BBG led to a significant reduction in seeding efficiency of neuroblastoma cells. According to the literature, it also caused a delay in tumor growth, reflected by smaller size and lower weight of tumors of BBG-treated mice, when compared to control mice (Ryu et al., 2011).

In summary, our findings indicate that kinin and purinergic signaling systems are important for dissemination and metastasis of neuroblastoma to the BM. Therefore, molecular strategies to inhibit those signaling pathway, together with the SDF-1/CXCR4 axis blockade, could lead to the development of novel more efficient anti-metastatic therapies to complement the conventional chemotherapy and radiotherapy in preventing the metastatic spread of neuroblastoma and other types of cancer.

## Author contributions

GS, EA, EO, EF, TG, JC-V, PM, FC, AS, MP, and CL performed the experiments; CL summarized the literature and drafted the manuscript; HU, EA, CL, and US revised and edited the manuscript. HU, CL, GS, and MR supervised the work; HU and CL initiated, finalized, and submitted the manuscript.

### Conflict of interest statement

The authors declare that the research was conducted in the absence of any commercial or financial relationships that could be construed as a potential conflict of interest.
